# Efficacy of selenium- and tellurium-based organocompounds against ruminant gastrointestinal nematodes *in vitro*

**DOI:** 10.1590/S1984-29612026017

**Published:** 2026-06-15

**Authors:** Taynara Gabriele Ribeiro Piano, Irineu Romero, Leandro Piovan, Leandro Bittencourt de Oliveira, Alda Lúcia Gomes Monteiro, Tay Zugman, Bernardo Ariel Schorr Zotz, Ursula Yaeko Yoshitani, Marcelo Beltrão Molento

**Affiliations:** 1 Universidade Federal do Paraná – UFPR, Departamento de Medicina Veterinária, Setor de Ciências Agrárias, Curitiba, PR, Brasil; 2 Universidade Federal do Paraná – UFPR, Departamento de Química, Setor de Ciências Exatas, Curitiba, PR, Brasil; 3 Universidade Federal do Paraná – UFPR, Departamento de Agronomia, Setor de Ciências Agrárias, Curitiba, PR, Brasil; 4 Universidade Federal do Paraná – UFPR, Departamento de Zootecnia, Setor de Ciências Agrárias, Curitiba, PR, Brasil

**Keywords:** Organoselenium compounds, organotellurium compounds, helminths, innovative therapy, drug-drug interaction, Compostos organoselenados, compostos organotelurados, helmintos, terapia inovadora, interação droga-droga

## Abstract

Gastrointestinal nematode (GIN) infections significantly impact the health of ruminants. The indiscriminate use of anthelmintics contributes to the selection of drug-resistant parasites. Compounds containing selenium (Se) and tellurium (Te) have emerged as promising alternatives. This study aimed to evaluate the anthelmintic activity of diphenyl diselenide (PhSe)_2_, phenylselenenyl chloride (4-Cl-PhSe)_2_, and ammonium trichloro (dioxoethylene-O,O’) tellurate (AS-101) on eggs and larvae (L3) of GIN. The compounds were tested using egg-hatch and larval-migration inhibition tests. Additionally, the possible *in vitro* cytotoxic effects were evaluated using the AlamarBlue assay. (PhSe)_2_ exhibited the highest ovicidal effect, with a 50% inhibitory concentration (IC_50_) of 1.801 mmol L^−1^ for GIN isolated from sheep and 1.845 mmol L^−1^ for GIN from cattle. AS-101 showed the highest larvicidal activity, with IC_50_ values of 0.976 mmol L^−1^ for GIN of sheep and 1.035 mmol L^−1^ for GIN of cattle. Combinations of (PhSe)_2_ with ivermectin (IVM) showed a mild synergistic effect, with an additive interaction of 13.33%. Among the tested compounds, (PhSe)_2_ showed the lowest cytotoxicity in LLC-MK2 cells. Taken together, these data highlight the therapeutic potential of the novel molecules.

## Resumo

As infecções por nematódeos gastrintestinais (NGI) afetam significativamente a saúde de ruminantes. O uso indiscriminado de anti-helmínticos contribui para a seleção de parasitos resistentes aos medicamentos. Compostos contendo selênio (Se) e telúrio (Te) surgiram como alternativas promissoras. Este estudo teve como objetivo avaliar a atividade anti-helmíntica do disseleneto de difenila (PhSe)_2_, do cloreto de fenilselenílio (4-Cl-PhSe)_2_ e do telurato triclorado de amônio (dioxietileno-O,O’) (AS-101) sobre ovos e larvas (L3) de NGI. Os compostos foram testados por meio dos testes de eclosão de ovos e de inibição da migração larval. Adicionalmente, os possíveis efeitos citotóxicos *in vitro* foram avaliados por meio do ensaio com AlamarBlue. O (PhSe)_2_ apresentou o maior efeito ovicida, com concentração inibitória de 50% (IC_50_) de 1,801 mmol L^-1^ para NGI isolados de ovinos e de 1,845 mmol L^-1^ para NGI de bovinos. O AS-101 apresentou a maior atividade larvicida, com IC_50_ de 0,976 mmol L^-1^ para NGI isolados de ovinos e de 1,035 mmol L^-1^ para NGI de bovinos. Combinações de (PhSe)_2_ com ivermectina (IVM) mostraram um efeito sinérgico leve, com interação aditiva de 13,33%. Entre os compostos testados, o (PhSe)_2_ apresentou a menor citotoxicidade em células LLC-MK2. Em conjunto, esses dados destacam o potencial terapêutico das novas moléculas.

## Introduction

Gastrointestinal nematode (GIN) infections are a major constraint on ruminant health and productivity, particularly in tropical and subtropical regions, resulting in substantial economic losses (Williams et al., 2021). These parasites most commonly cause subclinical infections characterized by reduced feed efficiency, weight gain, and milk production. In more severe cases, clinical signs such as anemia, lethargy, and anorexia may occur, negatively impacting animal welfare (Flay et al., 2022). The genera most commonly implicated in GIN infections among ruminants include *Haemonchus*, *Trichostrongylus*, *Teladorsagia* (formerly *Ostertagia*), and *Oesophagostomum*, with *Haemonchus contortus* recognized as the most pathogenic species (López-Rodríguez et al., 2023). As a result, the economic impact of GIN infections is substantial, leading to reduced animal productivity and substantial financial losses in the livestock industry (Chagas et al., 2022).

Parasite control relies on the use of broad-spectrum anthelmintics, including benzimidazoles, imidazothiazoles, and macrocyclic lactones (Evans & Sargison, 2019). Among these, ivermectin (IVM) exerts its anthelmintic effect by binding to glutamate-gated chloride channels in neuronal and muscle cells, leading to parasite paralysis and death (Laing et al., 2017). Moreover, IVM has been identified as a modulator of P-glycoprotein (P-gp), suggesting its potential role as an agent capable of reversing multidrug resistance (MDR) mechanisms (Rodrigues et al., 2025). However, its inappropriate use has led to the development of anthelmintic resistance (Gainza et al., 2021). Additionally, anthelmintic drugs can enter animal-derived food products and the environment, leading to the accumulation of persistent residues. These residues may disrupt ecological balance and contribute to the emergence of anthelmintic resistance. (Mesfin et al., 2024; Saeed et al., 2024).

The therapeutic potential of selenium- and tellurium-containing organochalcogens (OCs) has gained increasing attention due to their broad spectrum of biological activities. As members of the chalcogen group, selenium and tellurium share similar chemical properties and play essential roles in redox-regulated enzymatic systems. These chalcogens can modulate oxidative stress responses and influence key cellular functions, thereby affecting parasite survival and host-pathogen interactions (Valente et al., 2024). While the antiparasitic efficacy of organoselenium compounds has been more documented (Doleski et al., 2017; Martín-Escolano et al., 2021), organotellurium compounds have been comparatively less explored. Nevertheless, both Se- and Te-containing OCs have demonstrated promising pharmacological properties, including antifungal (Munhoz et al., 2023), antimicrobial (Borges et al., 2021), and immunomodulatory (Mishra et al., 2019) activities.

Our group has explored the antiprotozoal activity of dichalcogenide compounds and Te-based organochalcogens (OCs) containing heterocycles against *Leishmania* parasites (Bandeira et al., 2019; Souza et al., 2021; Valente et al., 2024). Additionally, we reported the first study to demonstrate the ovicidal activity of diaryl dichalcogenides against *Fasciola hepatica* eggs (Romero-Neto et al., 2024). Furthermore, we observed that combining Te- and Se-containing OCs with IVM resulted in a synergistic effect, enhancing larval migration inhibition by 10% to 34% (Romero-Neto et al., 2025). These findings further underscore the potential of such compounds as promising candidates for the development of novel therapeutic strategies against parasitic infections.

Therefore, the present study aimed to evaluate the anthelmintic efficacy of diphenyl diselenide (PhSe)_2_, phenylselenyl chloride (4-Cl-PhSe)_2_, and ammonium trichloro (dioxoethylene-O,O') tellurate (AS-101), both as individual agents and in combination with IVM, against gastrointestinal nematodes affecting ruminants.

## Material and Methods

### Chemical compounds

**Figure 1:**
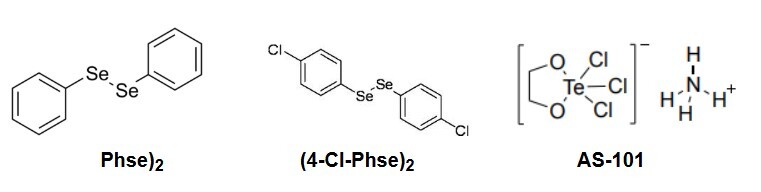
Chemical structure of diphenyl diselenide (PhSe)_2_, phenylselenyl chloride (4-Cl-PhSe)_2_, and ammonium trichloro (dioxyethylene-O,O') tellurate (AS-101).

Diphenyl diselenide (PhSe)_2_ and phenylselenyl chloride (4-Cl-PhSe)_2_ were synthesized according to Paulmier (1986), and ammonium trichloro (dioxyethylene-O,O') tellurate (AS-101) was synthesized following Albeck et al. (1989) (Figure 1). Spectroscopic data (^1^H, ^13^C, ^77^Se and ^125^Te NMR and FTIR) of all synthesized compounds were confirmed by the Nuclear Magnetic Resonance (NMR) and Fourier Transform Infrared Spectroscopy techniques (data not shown). The compounds were precisely weighed and dissolved in distilled water containing 0.1% DMSO to prepare the working solutions. Serial dilutions were then prepared from these stock solutions up to 1 h before each assay. All solutions were thoroughly vortexed to ensure complete dissolution.

Dimethyl sulfoxide (DMSO) and methanol were obtained from Hexis Científica (Jundiaí, Brazil). Pure reference standards of IVM and RPMI 1640 medium were obtained from Sigma-Aldrich (St. Louis, USA). Fetal bovine serum (FBS), penicillin, and streptomycin were acquired from Gibco Life (Grand Island, USA). AlamarBlue was obtained from Thermo Fisher Scientific (Waltham, USA).

### Recovery of nematode eggs and third-stage larvae (L3)

Fecal samples were collected directly from the rectum of naturally infected cattle and sheep from the Agricultural Innovation Center (NITA) and the Sheep and Goat Research Laboratory (LAPOC) of UFPR. The samples were processed separately according to host species, and the isolated parasites were tested in independent experiments to account for host-specific differences.

Egg recovery was carried out according to the method described by Coles et al. (1992) with modifications. Feces were homogenized and passed through a series of sieves (250, 150, 75, 38, and 25 μm) for filtration (Bertel Ltda, Caieiras, Brazil). The eggs were decanted and separated by centrifugation (Parsec Biotech, Penha, Brazil) at 3,000 rpm (≈ 1,000 × g) for 5 min using a saturated sodium chloride solution. The eggs were then collected using a 25 μm sieve and washed with tap water. L3 were recovered after coproculture following Roberts & O'Sullivan (1950). For coproculture, feces were mixed with vermiculite, moistened with distilled water, and placed in glass jars for culturing. The cultures were incubated in a BOD incubator (S.S. Santana, Londrina, Brazil) at 27°C for 10 days, with a relative humidity of 80%. After incubation, tap water at 40°C was added until the jar was full, and then quickly inverted over a Petri dish. Twelve milliliters of water were added to the Petri dish, and after 12 h, the contents were collected using a Pasteur pipette and transferred to a test tube. Larval identification was performed based on morphological characteristics using a light microscope, as described by van Wyk & Mayhew (2013).

The *H. contortus* isolated from sheep used in this study has been continuously monitored since 2005 through the fecal egg count reduction test (FECRT), revealing a consistent pattern of resistance to IVM, with an average efficacy of only 54.9% over the years (M.C. Cintra, personal communication, 2022). The susceptible/resistant status of parasites isolated from cattle remains limited.

### Egg Hatch Test (EHT)

EHT was conducted according to Dolenga et al. (2023) with modifications. Nematode eggs were distributed into 24-well plates (200 eggs/well) and treated with the compounds at the following concentrations: 0.10, 0.25, 0.50, 1.00, 2.00, 4.00, 8.00, and 16.00 mmol L^-1^. Controls included 50% DMSO (positive) and distilled water with 0.1% DMSO (negative). All plates were incubated in a BOD chamber (Quimis Ltda, Diadema, Brazil) for 48 h at 27°C. After incubation, 6 μL of Lugol’s iodine solution was added to each well to halt egg hatching. The plates were examined under an inverted microscope (Optiphase INV-403, Van Nuys, USA). The inhibition of egg hatchability percentage was calculated according to the formula (Dolenga et al., 2023):


$$\;Percent\;hatchability\left(\%\right)\;=[Eggs/\;\left(eggs\;+\;L1\right)]\times \;100$$ (1)

Where L1 corresponds to the first-stage larvae, the efficacy was assessed by counting the number of eggs initially present and the number of L1 after treatment.

### Larval Migration Inhibition Test (LMIT)

LMIT was performed as described by Romero-Neto et al. (2025). Fresh L3 were exsheathed with 1% (v/v) sodium hypochlorite, washed three times by centrifugation (2,500 rpm, ≈ 700 × g for 5 min), and quantified under a light microscope (Kasvi Ltda-Motic Instruments, Texas, USA) at 100x magnification. Approximately 100 L3 per well were incubated in 15 mL Falcon tubes for 24 h at 28°C and 80% relative humidity in a BOD incubator (Quimis Ltda, Diadema, Brazil). The compounds were tested at the following concentrations: 0.10, 0.25, 0.50, 1.00, 2.00, 4.00, 8.00, and 16.00 mmol L^-1^. IVM was also tested at concentrations of 0.10, 0.25, 0.50, 0.75, 1.00, 2.00, and 3.00 mmol L^-1^. Controls included 10% DMSO (positive) and distilled water with 0.1% DMSO (negative). After incubation, the entire content of each tube was transferred to a 24-well plate containing a 25 μm mesh/well for 24 h incubation under the same conditions. The reading was performed using an inverted light microscope (Optiphase INV-403, Van Nuys, USA) by quantifying the number of L3 that migrated through the mesh. The mean number of migrated L3 was calculated using the formula adapted from Molento & Prichard (2001):

### $Efficacy\;\left(\%\right)\;=\;\left(B\;-\;A\;/\;B\right)\;\times \;100$(2)

Where B is the negative control (distilled water), and A is the mean number of L3 that migrated after incubation.

### Drug combination assay

Inhibitory concentration values (IC_10_, IC_30_, and IC_50_) were calculated for each compound, and the fixed IC_50_ value was used for the drug combination assay. Dilutions were prepared using the fixed IC_50_ of [PhSe)_2_] combined with [IVM] at IC_10_, IC_30_, and IC_50_, and the fixed IC_50_ of [IVM] combined with [PhSe)_2_] at IC_10_, IC_30_, and IC_50_. The same procedure was applied for AS-101. The LMIT was performed, and L3 were quantified using an inverted light microscope (Optiphase INV-403, Van Nuys, USA).

### Cell viability by the AlamarBlue assay

LLC-MK2 cells were seeded at 5 × 10^4^ cells per well in 96-well microplates for 24 h at 37°C in a humidified incubator with 5% CO_2_. After incubation, cells were treated with (PhSe)_2_ at concentrations ranging from 4.0 to 0.5 mmol L^-1^ and 1.0 to 0.05 mmol L^-1^ for AS-101, for 24 and 48 h under the same conditions in RPMI 1640 supplemented with 10% heated-inactivated FBS, 1% antibiotics (100 U mL^-1^ of penicillin and 100 µg mL^-1^ of streptomycin), and 0.1% DMSO. After treatment, cells were washed once with PBS and incubated with 100 μL of 10% AlamarBlue for 2 h under the same conditions. Fluorescence was measured using a Varioskan LUX multimode microplate reader (Thermo Fisher Scientific, Vantaa, Finland) at 560 nm (excitation) and 590 nm (emission) (Barreiro et al., 2022). Cells treated with methanol (32 mol L^-1^) were used as a positive control. Untreated cells incubated in medium supplemented with 0.1% DMSO served as a negative control.

### Statistical analysis

Data were presented as the mean of three independent experiments performed in triplicate. The IC_10_, IC_30_, and IC_50_ values were estimated using nonlinear regression. Results were evaluated by one-way analysis of variance (ANOVA), followed by Dunnett’s post-hoc test (Romero-Neto et al., 2025). Synergistic effects were determined using the Synergistic Toxicity Profiler (SynToxProfiler), as proposed by Ianevski et al. (2020). Differences were considered statistically significant at P ≤ 0.05. Statistical analyses were performed using GraphPad Prism version 8.0.2 (San Diego, USA).

## Results

### L3 composition

*Haemonchus* spp. were the predominant L3 in the sheep samples, accounting for 76% of the nematode population, followed by *Trichostrongylus* spp. (16%). *Oesophagostomum* spp. and *Cooperia* spp. were less prevalent (6 and 2%, respectively). In the bovine samples, *Haemonchus* spp. It was also the most abundant (64%), followed by *Trichostrongylus* spp. (24%), *Oesophagostomum* spp. (9%), and *Cooperia* spp. (3%).

### Egg Hatch Test (EHT)

All tested compounds exhibited ovicidal activity, achieving 100% efficacy at the highest concentration. In the EHT using ovine parasites, (PhSe)_2_ demonstrated the greatest effectiveness, with the lowest IC_50_ value of 1.801 mmol L^-1^. In contrast, (4-Cl-PhSe)_2_ exhibited the highest IC_50_ of 2.267 mmol L^-1^, while AS-101 showed an intermediate IC_50_ of 1.961 mmol L^-1^.

**Figure 2:**
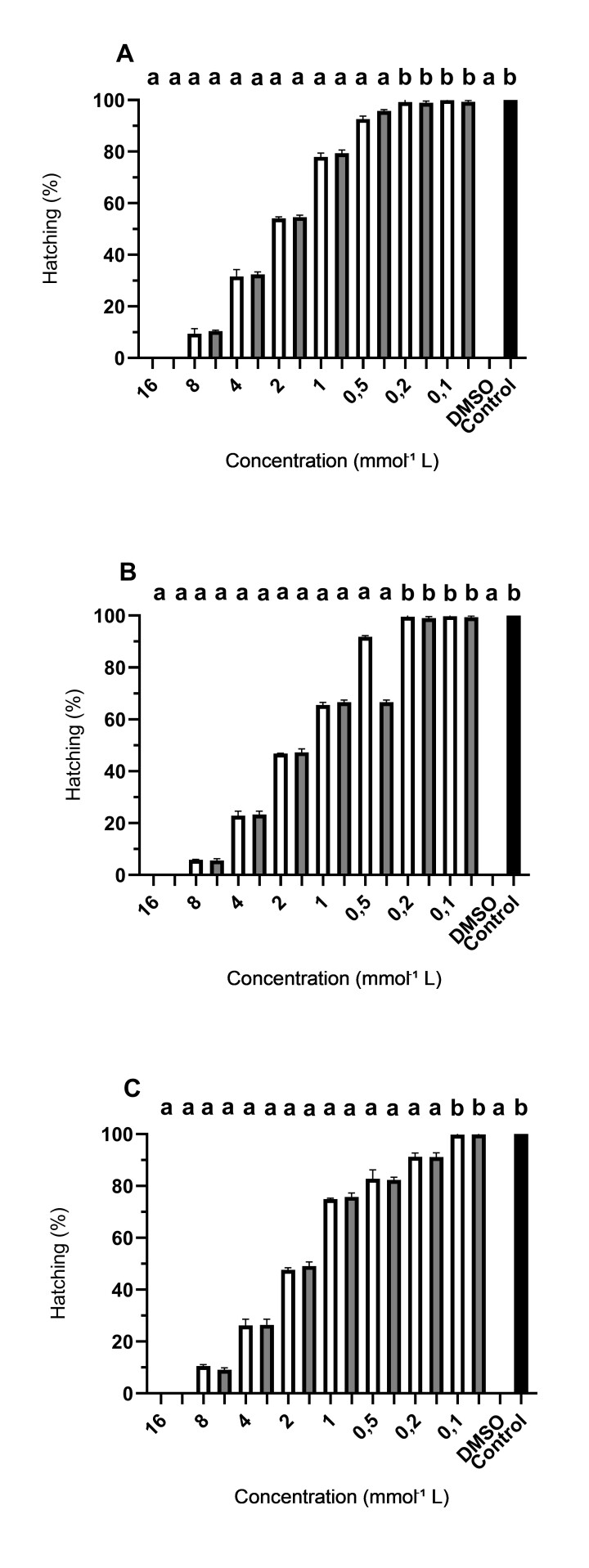
Percentage of egg hatching of gastrointestinal nematodes of sheep (white bars) and cattle (gray bars) treated with (A) phenylselenyl chloride (4-Cl-PhSe)_2_, (B) diphenyl diselenide (PhSe)_2_, and (C) ammonium trichloro (dioxyethylene-O,O') tellurate (AS-101). Data on ovicidal activity are expressed as mean ± standard deviation (SD). Significant statistical differences between the concentrations of each compound are indicated by different letters (P ≤ 0.05; ANOVA followed by Dunnett’s post-hoc test).

Similarly, in the EHT with bovine parasites, (PhSe)_2_ maintained the lowest IC_50_ value (1.845 mmol L^-1^), followed by AS-101 (2.029 mmol L^-1^), and (4-Cl-PhSe)_2_ (2.414 mmol L^-1^). Overall, the compounds demonstrated ovicidal activity across both host species (Figure 2).

### Larval Migration Inhibition Test (LMIT)

**Figure 3:**
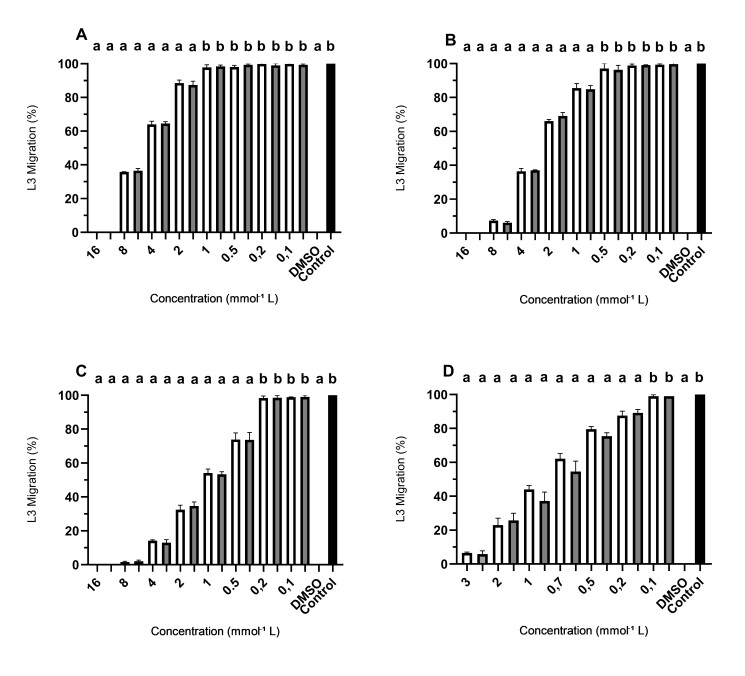
Migration of third-stage larvae (mean ± standard deviation, SD) of gastrointestinal nematodes of sheep (white bars) and cattle (gray bars) treated with (A) phenylselenyl chloride (4-Cl-PhSe)_2_, (B) diphenyl diselenide (PhSe)_2_, (C) ammonium trichloro (dioxyethylene-O,O') tellurate (AS-101), and (D) ivermectin (IMV). Significant statistical differences between the concentrations of each compound are indicated by different letters (P ≤ 0.05; ANOVA followed by Dunnett’s post-hoc test).

Data from the LMIT revealed a concentration-dependent response for the compounds, with the highest concentration resulting in 100% efficacy. Regarding the 50% inhibitory concentration (IC_50_) against ovine nematodes (Figure 3), (PhSe)_2_ and AS-101 exhibited greater efficacy compared to (4-Cl-PhSe)_2_, with IC_50_ values of 2.332 mmol L^-1^ and 0.976 mmol L^-1^, respectively. In contrast, (4-Cl-PhSe)_2_ presented a higher IC_50_ value of 4.528 mmol L^-1^.

In the LMIT conducted with bovine parasites (Figure 3), (PhSe)_2_ exhibited an IC_50_ of 2.503 mmol L^-1^, while (4-Cl-PhSe)_2_ showed a higher IC_50_ of 4.304 mmol L^-1^. AS-101 demonstrated superior potency among the organochalcogens, with an IC_50_ of 1.035 mmol L^-1^. IVM remained the most effective compound overall, displaying the lowest IC_50_ value of 0.819 mmol L^-1^ against ovine larvae and 0.760 mmol L^-1^ against bovine larvae.

The IC_50_ values of the tested compounds and IVM in L3 migration are summarized in Table 1. The data obtained for the compounds were used in the drug combination assay.

**Table 1:** Inhibitory concentration (IC) of AS-101, (PhSe)_2_, 4-Cl-(PhSe)_2_, and IVM against L3 nematodes from sheep and cattle.

	Sheep				Cattle			
IC	AS-101	(PhSe)_2_	(4-Cl-PhSe)_2_	IVM	AS-101	(PhSe)_2_	(4-Cl-PhSe)_2_	IVM
90	4.366	9.635	14.700	2.525	4.705	9.111	15.740	2.419
70	1.740	4.030	7.490	1.265	1.855	4.119	7.096	1.188
50	0.976	2.332	4.528	0.819	1.035	2.503	4.304	0.760
30	0.548	1.349	2.737	0.530	0.575	1.521	2.610	0.486
10	0.218	0.564	1.227	0.265	0.227	0.687	1.177	0.239

Obs. Data are expressed as mmol L^-1^. The Inhibitory Concentration (IC) values were estimated using nonlinear regression.

### Drug combination assay

The results of the combinations of IVM IC_50_ with (PhSe)_2_ and AS-101 revealed a significant interaction effect on L3 of both ovine and bovine hosts (Table 2). At certain combinations, such as IVM IC_50_ with (PhSe)_2_ IC_10_, an efficacy increase of up to 13.33% was observed, indicating a mild synergistic effect.

**Table 2:** Larvicidal activity (%) and additive interaction (%) of combinations of different inhibitory concentrations (CI) of ivermectin (IVM) with (PhSe)_2_ and AS-101 against larvae of nematodes of sheep and cattle.

Combination	Larvicidal activity (SD)	Additive interaction
Sheep		
IVM50 + (PhSe)_2_50	92.65 (± 1.36)	-7.35
IVM50 + (PhSe)_2_30	86.38 (± 1.35)	+6.38
IVM50 + (PhSe)_2_10	73.33 (± 1.34)	+13.33
(PhSe)_2_50 + IVM30	85.18 (± 1.42)	+5.18
(PhSe)_2_50 + IVM10	58.67 (± 3.53)	-1.33
IVM50 + AS-10150	88.97 (± 2.29)	-11.03
IVM50 + AS-10130	82.04 (± 2.10)	+2.04
IVM50 + AS-10110	64.23 (± 1.66)	+4.23
AS-10150 + IVM30	80.79 (± 1.98)	+0.79
AS-10150 + IVM10	66.65 (± 2.26)	+6.65
Cattle		
IVM50 + (PhSe)_2_50	91.55 (± 0.49)	-8.45
IVM50 + (PhSe)_2_30	86.54 (± 1.88)	+6.54
IVM50 + (PhSe)_2_10	69.08 (± 2.00)	+9.08
(PhSe)_2_50 + IVM30	86.55 (± 1.25)	+6.55
(PhSe)_2_50 + IVM10	68.04 (± 2.37)	+8.04
IVM50 + AS-10150	84.29 (± 1.33)	-15.71
IVM50 + AS-10130	75.56 (± 1.28)	-4.44
IVM50 + AS-10110	65.70 (± 1.14)	+6.70
AS-10150 + IVM30	81.92 (± 1.34)	+1.92
AS-10150 + IVM10	69.05 (± 1.35)	+9.05

Obs. Data are presented as mean ± standard deviation (SD) based on three experiments. Synergistic effects were determined using the Synergistic Toxicity Profiler (SynToxProfiler).

In general, combinations involving IC_10_ concentrations of the OCs yielded the most favorable results, suggesting a slight synergistic effect at subinhibitory levels. However, most combinations showed no additional efficacy. Combining IVM IC_50_ with AS-101 IC_50_ showed an antagonistic interaction against L3 parasites.

### Cell viability by the AlamarBlue assay

**Figure 4:**
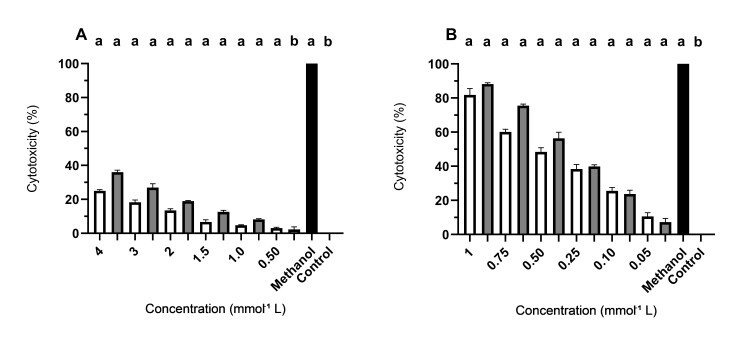
Cytotoxicity (%) (mean ± standard deviation, SD) of LLC-MK2 cells following treatment with (A) diphenyl diselenide (PhSe)_2_ and (B) ammonium trichloro (dioxoethylene-O,O’) tellurate (AS-101) for 24 (white bars) and 48 hours (gray bars). Significant statistical differences between the concentrations of each compound are indicated by different letters (P ≤ 0.05; ANOVA followed by Dunnett’s post-hoc test).

A time- and concentration-dependent cytotoxic effect was observed, with lower cytotoxicity at lower concentrations (Figure 4). AS-101 exhibited cytotoxicity at higher concentrations, reaching cell death of 81.80% at 24 h and 88.80% at 48 h. In contrast, (PhSe)_2_ demonstrated significantly lower cytotoxicity, causing 25.80% cell death at 24 h and 36.05% at 48 h. Based on these findings, the CC_50_ values for AS-101 were 0.363 mmol L^-1^ at 24 h and 0.307 mmol L^-1^ at 48 h. For (PhSe)_2_, the CC_50_ values were 2.166 mmol L^-1^ at 24 h and 1.959 mmol L^-1^ at 48 h, indicating a more favorable cytotoxicity profile.

## Discussion

The results from both EHT and LMIT demonstrate concentration-dependent inhibition of egg hatchability and larval migration in gastrointestinal nematodes of ruminants by all tested compounds. Within the EHT, (PhSe)_2_ emerged as the most effective ovicidal agent, whereas in the LMIT, AS‑101 exhibited higher efficacy at lower concentrations. Although (4‑Cl‑PhSe)_2_ also displayed inhibitory activity, it was less potent in the LMIT, requiring concentrations 3x to 4x times higher than those of AS‑101 and (PhSe)_2_ to achieve similar IC_50_ values.

These variations in efficacy can be attributed to differences in chemical structure and reactivity. According to Piovan et al. (2011), Te-containing compounds are inherently more electrophilic than organoselenium analogs, facilitating rapid binding to nucleophilic biomolecular targets. The superior ovicidal performance of (PhSe)_2_ in the EHT may stem from its ability to penetrate the structural layers of eggs, bind to tubulin, and inhibit essential developmental proteins (Santos et al., 2013; Tang et al., 2021). On the other hand, the Se-Cl bond in (4‑Cl‑PhSe)_2_ is more electrophilic but less stable, potentially reducing its reactivity and binding efficiency to biological targets.

Furthermore, AS‑101 outperformed the Se-based compounds in the LMIT. This enhanced efficacy may reflect Te's ability to inhibit cathepsin B, a cysteine protease crucial to *H. contortus* survival. *H. contortus* expresses cathepsin B-like proteases essential for hemoglobin and fibrinogen degradation (Bakshi et al., 2021). Te‑ and Se‑containing compounds have also been shown to inhibit cysteine proteases involved in embryogenesis, molting, hatching, and larval motility (Piovan et al., 2010; Capper et al., 2018; Grote et al., 2018). Piovan et al. (2011) reported approximately 90% inhibition of cathepsins V and S by Te-containing organocompounds and 70-80% inhibition by Se-containing organocompounds at 1 μmol L^-1^.

In a previous study, Se-containing OCs induced widespread propidium iodide (PI) labeling in GIN L3, indicating extensive cell death across multiple tissues and organs. In contrast, treatment with LQ07, an organotelluride compound, resulted in a more restricted PI labeling pattern, indicating that the intestinal epithelium and neural structures, such as the anterior and posterior nerve rings, were the most susceptible targets (Romero-Neto et al., 2025).

When evaluated individually, IVM exhibited the most potent anthelmintic effect against GIN eggs and L3 compared to the tested OCs. However, significant effects were observed only at high IVM concentrations. At these elevated concentrations, drug precipitation may occur, potentially leading to nonspecific physical interactions with the parasites. It is important to note that the concentrations used in this *in vitro* study are substantially higher than those typically achieved *in vivo*. Thus, a direct correlation between *in vitro* efficacy and *in vivo* pharmacokinetics is not possible. Although IVM showed modest ovicidal activity and larval migration inhibition *in vitro*, these effects do not reflect its *in vivo* efficacy profile, in which the compound is known to lack activity against nematode eggs and to exhibit larvicidal activity at lower concentrations (Demeler et al., 2010). Such discrepancies underscore the limitations of *in vitro* assays and the need for caution when extrapolating results to physiological conditions.

Nevertheless, the novel combination of (PhSe)_2_ and AS‑101 with IVM aimed to enhance therapeutic efficacy and address multidrug resistance. These combinations exhibited a mild synergistic effect (additive range: 0.79-13.33%) in inhibiting L3 migration, a finding that parallels those reported with other pathogens (Bortoluzzi et al., 2021; Gnat et al., 2022; Munhoz et al., 2023). Although the synergism analysis indicated a mild synergistic effect (up to 13.3%), this level of interaction is considered weak and may lack practical relevance. Therefore, the observed synergy should be interpreted with caution. Further investigations using different concentration ratios or alternative compound pairings are warranted to explore more potent synergistic interactions.

Despite the scarcity of previous research specifically addressing the anthelmintic activity of (PhSe)_2_, (4‑Cl‑PhSe)_2_, and AS‑101 against GIN of ruminants, diaryl dichalcogenides have demonstrated promising efficacy *in vitro* against gastrointestinal nematodes of sheep (Romero-Neto et al., 2025) and *F. hepatica* (Romero-Neto et al., 2024), with a synergistic effect on L3 larval migration inhibition with additive interaction ranging from 10.7% to 33.9% and 1.1% to 27.5%, respectively. Moreover, AS‑101 has shown *in vivo* and *in vitro* antibacterial activity against carbapenem-resistant *Pseudomonas aeruginosa* (Li et al., 2023). However, experimental data concerning (4‑Cl‑PhSe)_2_ remain limited.

Toxicity profiling in LLC‑MK2 cells revealed that (PhSe)_2_ had an IC_50_ of 2.166 mmol L^-1^, approximately 6x higher than that of AS‑101 (0.363 mmol L^-1^ at 24 h), indicating a more favorable safety profile. These *in vitro* findings are consistent with previous reports demonstrating AS-101's selective safety. For instance, macrophages and Vero cells maintained >98% viability up to 100 μM, and even at 400 μM after 72 h, cytotoxicity did not exceed 22%, indicating a broad therapeutic window (Vishwakarma et al., 2018). Moreover, Yang et al. (2021) reported that AS-101 significantly reduced bacterial burden and improved survival in mice infected with carbapenem-resistant *Acinetobacter baumannii*, using doses well below its LD_50_ (10 mg/kg). Similarly, (PhSe) _2_ has shown protective effects in multiple models of infection and inflammation, significantly reducing oxidative stress markers, histological damage, and pro-inflammatory mediators at low doses (e.g., 5 mg kg^-1^ for 10 days) (Sartori et al., 2016; Sartori et al., 2017). Additionally, blood samples from (PhSe)_2_-supplemented sheep revealed enhanced antioxidant defenses and elevated levels of the anti-inflammatory cytokine IL-10 with no signs of systemic toxicity. Supplementation also led to increased milk fat content and reduced total protein and lactose levels (Biazus et al., 2019). Collectively, these findings highlight a favorable balance between efficacy and safety for both compounds, supporting their potential for therapeutic development.

Nevertheless, the toxic effects of Se- and Te-based organocompounds are likely concentration-dependent and may involve enzyme inhibition through interactions with thiol or selenol groups, leading to increased lipid peroxidation and DNA damage (Puntel et al., 2010; Comparsi et al., 2012). Although Se-containing OCs are described as antioxidant active agents, organoselenium and organotellurium compounds can trigger ROS-mediated mitochondrial depolarization and apoptosis (Nogueira et al., 2021; Valente et al., 2024). Moreover, organoselenium compounds may also trigger mitochondrial Ca^2+^ release via NAD^+^ hydrolysis, thereby accelerating respiration and inducing mitochondrial swelling (Azad et al., 2014).

Although none of the combinations achieved complete inhibition of L3 migration, the drug-drug interactions observed when combining Se- and Te-containing OCs with IVM against L3 suggest a novel therapeutic strategy. The combination of IVM with each tested compound may offer advantages due to their distinct mechanisms of action and ability to target independent sites within the parasite. Notably, organoselenium compounds have been identified as potent inhibitors of multidrug resistance (MDR) efflux pumps (Gajdács et al., 2017; Spengler et al., 2019), which may affect the bioavailability of drugs that are substrates of P-glycoprotein.

Although some of the concentrations tested, particularly in the millimolar range, are unlikely to be achieved *in vivo*, they are commonly used in early-stage *in vitro* screening to assess biological activity and establish dose-response relationships. These findings provide a valuable starting point for identifying promising candidates for further development, including studies focused on pharmacokinetics, safety, and *in vivo* efficacy. Altogether, the present findings contribute to the discovery of new properties of OCs and support their potential as scaffolds for the development of innovative chemotherapeutic agents against parasitic infections.

## Conclusion

The findings of this study demonstrate the *in vitro* efficacy of (PhSe)_2_, (4-Cl-PhSe)_2_, and AS-101 against eggs and L3 of GIN of ruminants. The compounds exhibited a concentration-dependent antiparasitic effect. The combination of IVM with (PhSe)_2_ resulted in a mild synergistic effect (13.3% at the lowest concentration). This study highlights the potential of Se- and Te-containing OCs as promising candidates for pharmacological innovation in the control of GIN in ruminants.

## Data Availability

The raw data supporting the results of this study are available upon request from the authors.
